# Navigating the metabolic maze: anomalies in fatty acid and cholesterol processes in Alzheimer’s astrocytes

**DOI:** 10.1186/s13195-024-01430-x

**Published:** 2024-03-23

**Authors:** Xiaoyu Zhang, Chuanying Chen, Yi Liu

**Affiliations:** 1 Department of Neurosurgery, Institute of Brain Diseases, Nanfang Hospital, Southern Medical University, Guangzhou, Guangdong 510515 China; 2 School of Traditional Chinese Medicine, Southern Medical University, 1838 North Guangzhou Avenue, Guangzhou, Guangdong 510515 People’s Republic of China

**Keywords:** Alzheimer’s disease, Astrocyte, Fatty acid, Cholesterol

## Abstract

Alzheimer’s disease (AD) is the most common cause of dementia, and its underlying mechanisms have been a subject of great interest. The mainstream theory of AD pathology suggests that the disease is primarily associated with tau protein and amyloid-beta (Aβ). However, an increasing body of research has revealed that abnormalities in lipid metabolism may be an important event throughout the pathophysiology of AD. Astrocytes, as important members of the lipid metabolism network in the brain, play a significant role in this event. The study of abnormal lipid metabolism in astrocytes provides a new perspective for understanding the pathogenesis of AD. This review focuses on the abnormal metabolism of fatty acids (FAs) and cholesterol in astrocytes in AD, and discusses it from three perspectives: lipid uptake, intracellular breakdown or synthesis metabolism, and efflux transport. We found that, despite the accumulation of their own fatty acids, astrocytes cannot efficiently uptake fatty acids from neurons, leading to fatty acid accumulation within neurons and resulting in lipotoxicity. In terms of cholesterol metabolism, astrocytes exhibit a decrease in endogenous synthesis due to the accumulation of exogenous cholesterol. Through a thorough investigation of these metabolic abnormalities, we can provide new insights for future therapeutic strategies by literature review to navigate this complex metabolic maze and bring hope to patients with Alzheimer’s disease.

## Background

Alzheimer’s disease (AD) is widely acknowledged as a highly fatal and burdensome ailment in contemporary times, serving as the primary instigator of dementia [1]. Despite extensive research into drugs targeting the reduction of amyloid-beta (Aβ) production or prevention of their aggregation, as well as drugs intended to impede inflammation, their therapeutic effectiveness remains less than optimal. Recent investigations into AD have indicated that the disruption of lipid metabolism significantly contributes to the development of the disease [2–4]. Over-expression of the familial amyloid precursor protein (APP) Swedish mutation affects lipid homeostasis in mitochondria-associated endoplasmic reticulum membranes (MAMs) and other subcellular fractions and supports the important role of lipids in AD physiopathology [5]. Remarkable advances in translational molecular imaging have now made it possible to probe cholesterol metabolism in the living human brain with positron emission tomography, which is an important prerequisite for future clinical trials that target the brain cholesterol machinery in AD patients [6]. Astrocytes, a widely distributed glial cell type in the central nervous system (CNS), participate in lipid signaling with neurons, oligodendrocytes, and microglia cells, and are primarily responsible for lipid synthesis and metabolism. The impairment of lipid metabolism in astrocytes is regarded as a fundamental occurrence in AD. This review seeks to clarify the significant role of lipid metabolism dysfunction in astrocytes in AD, specifically emphasizing the complete process of fatty acids (FAs) and cholesterol metabolism dysfunction in astrocytes, encompassing uptake, degradation, synthesis and release.

## Main text

### AD

AD serves as the primary cause of dementia, accounting for 60–70% of all instances. Furthermore, the prevalence of this ailment increases as individuals age [3]. The estimated number of people living with dementia globally was reported to be 57.4 million in 2019 and is expected to reach 153 million by 2050 [7].

In individuals afflicted with AD, there are discernible pathological modifications that transpire within cells as well as outside of cells. The intracellular build-up of hyperphosphorylated tau protein gives rise to the development of neurofibrillary tangles, whereas the extracellular build-up of Aβ protein gives rise to the appearance of Aβ plaques. The prevailing viewpoint in the realm of pathophysiology posits that the etiology of AD is intricately interconnected with Aβ and tau.The presence of Aβ and tau proteins, which deviate from the norm, initiates a series of subsequent occurrences, including inflammation and disturbances in cellular pathways such as lipid and glucose metabolism. The excessive production of Aβ or the hindrance of its removal has been associated with a heightened susceptibility to AD.

In the early stages of AD, prospective individuals manifest elevated levels of hyperphosphorylated tau in their cerebrospinal fluid, a phenomenon that disrupts microtubules and subsequently affects axonal transport and neural transmission [8], thereby contributing to cognitive decline. Synapses also play a significant role in the pathology of AD. Increasing evidence suggests that synaptic loss is an early disease process caused by the accumulation of soluble Aβ and phosphorylated tau protein, as well as increased production of free radicals by synaptic mitochondria. Moreover, extracellular Aβ aggregates at both postsynaptic spines and presynaptic spines, with a higher abundance observed at postsynaptic terminals. Further damage to glutamate transport can lead to dendritic spine loss [9]. Additionally, extracellular tau is involved in long-term plasticity by serving as a substrate for Glycogen Synthase Kinase 3β and p38 Mitogen-Activated Protein Kinase, further regulating synaptic function [10].

**Fig. 1:**
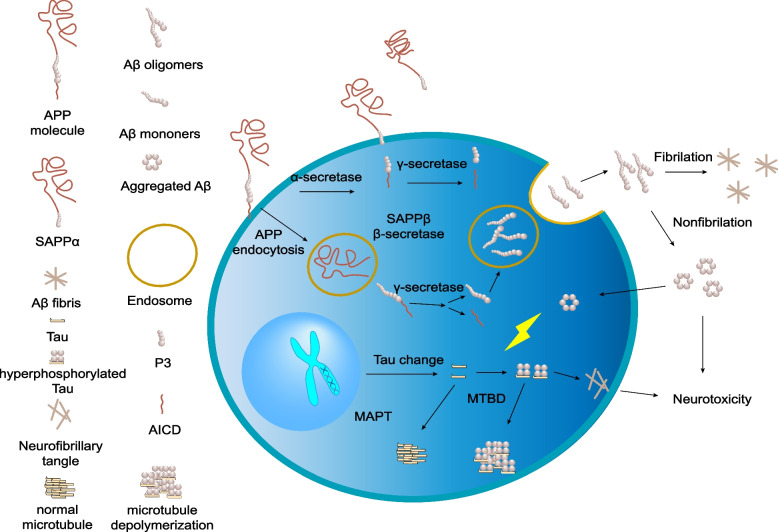
The transcription of the amyloid precursor protein (APP) gene leads to the cleavage of APP by α- and γ-secretases in the non-amyloidogenic pathway (plasma membrane) or by β- and γ-secretases in the amyloidogenic pathway (endosomal/lysosomal system), resulting in the extracellular release of amyloid beta (Aβ) peptide. Aβ monomers have the potential to form Aβ oligomers, which can fibrillize into Aβ fibrils under normal physiological conditions. However, under pathological conditions associated with Alzheimer’s disease (AD), Aβ aggregates can induce Tau-dependent neurotoxicity. The transcription and translation of the MAPT gene results in the production of Tau protein, which undergoes diverse post-translational modifications (PTMs) in various forms within the cell. In normal physiological conditions, the microtubule-binding domain (MTBD) of Tau binds to microtubules, thereby stabilizing them. However, in the presence of AD pathology, Tau is hyperphosphorylated, a process that can be influenced by Aβ aggregates, resulting in a reduction in the affinity of Tau for microtubules and rendering them unstable. Furthermore, hyperphosphorylated Tau can form neurofibrillary tangles in the cytoplasm, leading to cellular toxicity

It is crucial to acknowledge that these two factors are not independent, as there exists evidence indicating that Aβ-induced neurotoxicity relies on the presence of tau [11]. Tau may contribute to Aβ-induced harm via two mechanisms. First, tau plays a physiological role in the disruption of neural network activity caused by diverse pathogenic triggers [12, 13]. Second, Aβ modifies the post-translational modifications (PTMs) or distribution of tau, thereby converting tau into an active agent in the development of Aβ-induced neuronal dysfunction [14] (Fig. 1).

Moreover, recent research indicates that disturbances in lipid metabolism may interact with tau and Aβ pathology, intensifying neuronal injury and cognitive deterioration in AD. Lipid metabolism is intricately connected to AD through various mechanisms such as neuroinflammation, oxidative stress, mitochondrial dysfunction, and impaired synaptic transmission [15, 16]. Additionally, lipid metabolism plays a role in the exacerbation of neuronal damage and cognitive decline in AD by influencing Aβ and Tau pathology [17]. Products of lipid peroxidation and inflammatory mediators resulting from impaired lipid metabolism have the potential to exacerbate tau hyperphosphorylation and amyloid beta aggregation, thereby perpetuating a detrimental cycle of neurodegeneration.

### Astrocytes in physiological and pathological conditions

Astrocytes, derived from radial glial cells situated in the subventricular zone of the brain, constitute the predominant, intricate, and extensively interconnected non-neuronal cell population within the CNS. By means of neurotransmitter-mediated communication, astrocytes can directly modulate the stability, functionality, and adaptability of neurons [18]. Astrocytes possess the ability to indirectly modulate synaptic function by facilitating the phagocytosis of synaptic proteins by microglia via the secretion of IL-33 [19]. Additionally, they contribute to the maintenance of cellular ion homeostasis by engaging in the elimination of synapses and extracellular K + from axons through the involvement of membrane Na + /K + -ATPase. The Na + /K + -ATPase α2 and β2 isoforms are among the highest expressed proteins in hippocampal astrocytes, and its ability to respond directly to elevated the K + concentration in the extracellular space and the associated membrane depolarization makes it uniquely poised toward facilitating K + clearance during activity-evoked K + transients in the extracellular space [20].

Blood–brain barrier (BBB) is centrally positioned within the neurovascular unit (NVU). Different cell types of NVU, including astrocytes and microglia, regulate BBB integrity, cerebral blood flow, and participate in angiogenesis and neurogenesis. Astrocytes secrete apolipoprotein E (ApoE) to signal pericytes via low-density lipoprotein receptor-related protein-1 (LRP1), which suppress the activation of cyclophilin A-matrix metalloproteinase 9 BBB-degrading pathway. LRP1 binds AD’s Aβ toxin and mediates its brain-to-blood clearance. LRP1 levels at the BBB are diminished in AD mouse models and AD patients’ brains contributing to Aβ accumulation in the brain and activation of BBB-degrading pathway [21]. The events within microglia–astrocyte interaction include direct contact, cytokine secretion, complement-mediated interaction, receptor regulation, and exocytosis. The ion channels and ATP-mediated Ca2 + conduction may be also involved [22].

In contemporary times, scholarly inquiries pertaining to astrocytes have shifted from examining epigenetic variability to performing physiological examinations, encompassing the assessment of Ca2 + activity, ion buffering, gap junction coupling, and the expression of glutamate receptors [23]. A notable aspect of interest in astrocyte research has been their metabolic processes, as astrocytes play a pivotal role in the production of neuronal glycogen, which serves as the primary energy reserve in the brain [24]. Additionally, the lactate generated by astrocytes can function as a substrate for oxidation and as a signaling molecule for neurons [25].

Changes in astrocyte function can potentially contribute to the progression of various pathologies in the cerebral region. Among these, multiple sclerosis (MS) is characterized by the presence of persistent demyelinating lesions, axonal atrophy, widespread and localized demyelination of gray matter, and neurodegeneration caused by the depletion of oligodendrocytes and myelin. The acceleration of MS development has been observed through the activation of astrocytes by Th1, Th17, and Th1-like Th17 inflammatory cytokines, resulting in the disruption of the BBB, recruitment of leukocytes, and impairment of communication between neurons and oligodendrocytes [26]. Epilepsy is distinguished by the occurrence of unpredictable recurrent seizures, which are attributed to hypersynchronized excitatory neuronal discharges. The dysregulation of astrocytic potassium channels, resulting in an imbalance of potassium levels and impaired glutamate uptake, plays a significant role in the pathogenesis of epilepsy [27]. Additionally, the overexpression of adenosine kinase in astrocytes, leading to enhanced adenosine clearance, is a pathological hallmark of temporal lobe epilepsy.

Astrocytes have been implicated in a range of neurodegenerative disorders, such as AD, Huntington’s disease (HD), Parkinson’s disease (PD), and amyotrophic lateral sclerosis (ALS). The potential association between astrocytes and these diseases can be attributed to their involvement in protein aggregation, dysregulation of calcium and ion homeostasis, synaptic transmission, and impairment of energy metabolism [28].

HD is distinguished by the presence of chorea, emotional disturbances, and a gradual decline in cognitive function. This neurodegenerative disorder is caused by the expansion of CAG repeat sequences in the huntingtin gene. HD astrocytes exhibit a lower expression of excitatory amino acid transporter 2 (EAAT2) mRNA and protein compared to normal controls [29, 30]. This reduction in EAAT2 levels leads to a decrease in glutamate uptake, resulting in chronic glutamate stimulation within the brain and subsequent neuronal degeneration [31]. Moreover, the presence of mitochondrial damage in astrocytes located in the striatum, along with the compromised mitochondrial bioenergetics and dynamics caused by the mutated huntingtin protein, can potentially worsen the process of neurodegeneration in HD patients [32]. This ultimately leads to disturbances in lipid metabolism within the brains of individuals affected by HD.

PD is characterized by the progressive loss of dopaminergic neurons in the substantia nigra pars compacta, accompanied by the abnormal accumulation of misfolded α-synuclein in structures known as Lewy bodies.

The transfer of fibrillar α-synuclein from neurons to astrocytes demonstrates a higher level of efficiency, whereas the efficacy of transfer from astrocytes to neurons is comparatively lower. Moreover, astrocytes exhibit a greater proficiency in the degradation of fibrillar α-synuclein than neurons, suggesting that astrocytes may exert neuroprotective effects by capturing and breaking down pathological α-synuclein [33].

ALS, a prevalent paralytic disorder, typically presents in adulthood and is characterized by the degeneration of motor neurons within the CNS. Previous studies have demonstrated a reduction in the expression of glutamate transporter GLT1 and lactate transporter monocarboxylate transporter 1 (MCT1) in astrocytes containing the ALS-associated mutant superoxide dismutase-1 (SOD1) [34].

The association between astrocytes and AD has been extensively examined, revealing the aggregation of astrocytes around and within Aβ plaques in the CNS of AD patients. This occurrence has the potential to initiate neuroinflammation through various signaling pathways [35].

### Physiology and pathology of lipid metabolism in brain

The human brain, being the second-largest organ rich in lipids, exhibits a noteworthy association between lipid metabolism encompassing FAs and cholesterol metabolism, and the maintenance of brain energy equilibrium, oxidative stress, and neuroinflammation.

**Fig. 2:**
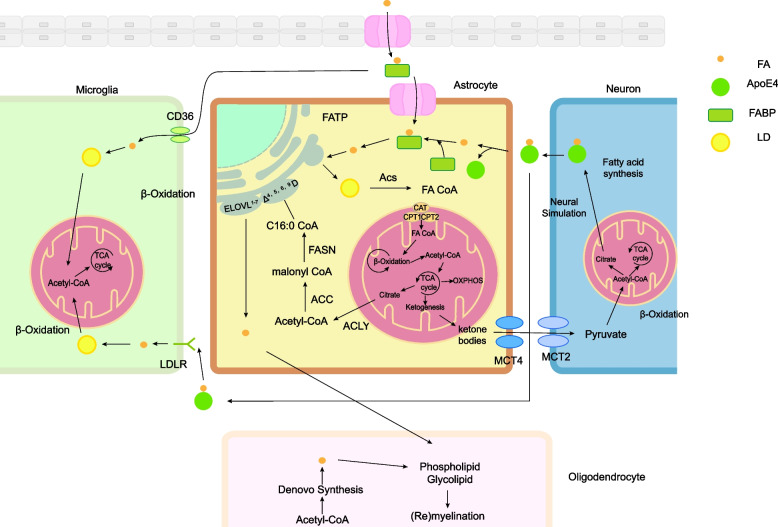
The brain’s fatty acid homeostasis is centred on astrocytes. The source of astrocyte fatty acids is the blood–brain barrier and neurons overproducing fatty acids in response to neural stimulation, which enter the cell via fatty acid transport protein (FATP), fatty acid binding protein (FABP) and apolipoprotein E, and form lipid droplets (LDs) for storage in the endoplasmic reticulum. Fatty acids are converted to fatty acyl-CoAs (FA-CoAs) by a family of acyl-CoA synthetases (ACS). Carnitine palmitoyl transferases (CPT) and carnitine-acylcarnitine translocases (CAT) translocate FA-CoAs into the mitochondrial matrix, where they participate in β-oxidation and the tricarboxylic acid (TCA) cycle. Fatty acid synthesis begins with the conversion of acetyl coenzyme A to malonyl coenzyme A, which is then extended to form C16:0-CoA, a reaction that requires the participation of acetyl coenzyme A carboxylases (ACCs), ATP citrate lyase (ACLY), and fatty acid synthase (FASN).Subsequent elongation and desaturation steps, catalysed by elongases (ELOVL1-7) and desaturases (Δ4,5,6,9D), form fatty acids of different carbon lengths and degrees of saturation. Ketone bodies, an intermediate product of fatty acid metabolism in astrocytes, can be transported via monocarboxylate transporter proteins (MCT) to supply energy to neurons. Microglia also perform part of the function of consuming excess fatty acids in the brain. Fatty acids produced by astrocytes may be transported to at least synaptic glial cells to participate in myelin formation or myelin regeneration

FAs enter astrocytes through specific transport proteins, becoming lipid droplets (LDs) stored in the endoplasmic reticulum. They are then converted into fatty acyl-CoAs, which are transported into the mitochondria to participate in energy-producing pathways like β-oxidation and the tricarboxylic acid (TCA) cycle. The synthesis of FAs involves converting acetyl coenzyme A into various FAs through a series of enzymatic reactions, leading to the production of molecules of different lengths and saturation. Astrocytes also produce ketone bodies for neuronal energy and may transport FAs to support myelin formation or regeneration, with microglia also aiding in managing excess FAs. Notably, LDs play a pivotal role in governing neurogenesis, synaptic function, and brain inflammation [36] (Fig. 2).

**Fig. 3:**
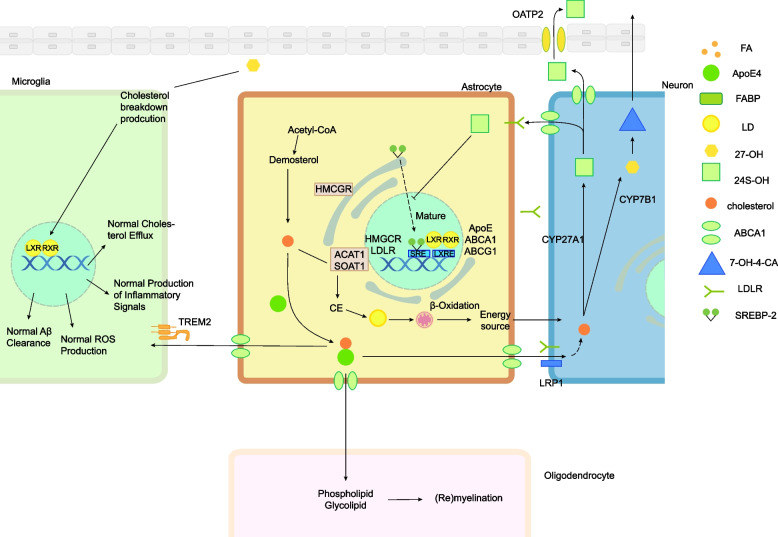
Brain cholesterol homeostasis is to a lesser extent related to peripheral tissues, as separated by the BBB barrier. The main pathways for cholesterol synthesis in the brain are the Bloch pathway in astrocytes, where cholesterol is derived from desmosterol, and to a lesser extent the Kandutsch-Russell pathway in neurons, where cholesterol is derived from 7-dehydrocholesterol. The synthesis process that is regulated by the sterol regulatory element-binding protein 2 (SREBP-2). In astrocytes, cholesterol binds to ApoE to form lipoproteins that are secreted into the extracellular fluid via ABC transporter proteins (especially ABCA1) and finally taken up and transported to neurons, microglia, and oligodendrocytes by the two types of LDL receptors, low density lipoprotein receptor (LDLR) and low-density lipoprotein receptor-related protein (LRP). ApoE is recycled after receptor-mediated endocytosis. Cholesterol is used for energy supply, normal production of inflammation-related signals and myelin formation. To maintain cholesterol homeostasis, excess cholesterol is esterified by enzyme acyl-coenzyme A: cholesterol acyltransferase 1 (ACAT1/SOAT1) in the andoplasmic reticulum and stored as cholesterol ester (CE) in LDs. The cholesterol associated with peripheral tissues by the brain is 24-hydroxycholesterol (24S-OH) and 27-hydroxycholesterol (27-OH). The neuron-specific enzyme 24-hydroxylase (CYP46A1) converts excess cholesterol to the more hydrophilic metabolite 24S-OH, which either diffuses into the somatic circulation via the BBB or acts as a A natural endogenous agonist of liver X receptor (LXR) controls cholesterol homeostasis. 27-OH is produced mainly in peripheral tissues by the catalytic activity of CYP 27 A1, which diffuses from the circulation to the brain via the BBB and is involved in microglia homeostasis. To a lesser extent, brain cholesterol is also oxidised to 27-OH by sterol 27-hydroxylase (CYP27A1), which is then oxidised by enzyme oxysterol 7-alpha-hydroxylase (CYP7B1) to 7α-hydroxy-3-oxo-4-cholestenoic acid (7-OH-4-C), which is cleared in the liver after passing through the BBB

Cholesterol is prominently concentrated within the brain, where it establishes lipid raft domains and facilitates the production of steroids, vitamin D and oxysterols [37]. Cholesterol synthesis in the brain primarily occurs through the Bloch pathway in astrocytes and the Kandutsch-Russell pathway in neurons. Astrocytes produce cholesterol that binds to ApoE and is secreted into the extracellular fluid, where it is taken up by neurons, microglia, and oligodendrocytes via LDL receptors. Cholesterol is essential for energy supply, inflammation signaling, and myelin formation. Excess cholesterol is stored as cholesteryl ester in LDs after esterification. The brain interacts with peripheral tissues through the conversion of cholesterol to more hydrophilic metabolites like 24-hydroxycholesterol (24S-OH) and 27-hydroxycholesterol (27-OH), which regulate cholesterol homeostasis within the brain and also contribute to peripheral cholesterol management. These metabolites can cross the BBB and are involved in various homeostatic processes, including microglial function and liver clearance (Fig. 3).

Notably, there is virtually no exchange of cholesterol with the peripheral circulation, due to the impermeable nature of the BBB, and thus cerebral cholesterol level is dependent on de novo synthesis by glial cells. On the contrary, certain cholesterol products with lipophilic properties, including 27-OH, can pass the BBB. Lipoproteins, including ApoE and lipoprotein lipase (LPL), have been found to have significant effects on intercellular communication, energy homeostasis, formation of the BBB, and regulation of pro- and anti-inflammatory responses. ApoE has received much attention due to the relationship of particular alleles of its gene with the risk and progression of AD. However, other lipid-binding proteins whose role in lipid homeostasis and control are less known need to be brought to the attention. ApoJ/Clusterin and ApoE are involved in the biological processes of BBB function, oxidative stress/inflammation and amyloidogenesis whereas ApoD is involved in the biological processes of oxidative stress/inflammation, lysosome, and myelin. ApoJ and ApoE transport cholesterol by linking directly to cholesterol, whereas ApoD is transported indirectly by linking to membranes [38].

Recent advancements in genome-wide association studies and transcriptomics research in the brain have revealed numerous abnormalities in lipid metabolism in neurological disorders. Notably, lipid metabolism disorders, particularly lipid peroxidation, have been linked to various neurodegenerative diseases. Significantly, mutations in the glucocerebrosidase gene increase the likelihood of inheriting PD, as glucocerebrosidase plays a crucial role in the metabolism of neuronal ceramides. Additionally, the APOE4 gene variant, a prominent genetic predisposition for AD, has been associated with cholesterol and sphingolipids [39].

The pathogenesis of several neurodegenerative disorders has been found to be closely linked to sphingolipids, which exhibit pro-apoptotic, autophagic, and inflammatory properties.

In the context of AD, it has been observed that extracellular vesicles containing sphingolipids stimulate the production of Aβ through interaction with lipid rafts. Lipid rafts are important platforms for various signaling molecules, including receptors, kinases, and adaptor proteins. Disruption of lipid rafts can lead to changes in signal cascades, affecting processes such as synaptic plasticity, neuronal survival, and inflammation. Stimulation of astrocytes with TNF-α results in the overexpression of MIP-2γ and downregulation of GLT-1 expression, leading to a redistribution of GLT-1-mediated glutamate uptake within lipid rafts [40]. These damaged signaling pathways may contribute to the development and progression of AD. APP is a transmembrane protein located on the cell membrane. Under normal conditions, it is involved in the growth, differentiation, and repair of neurons. However, in AD, APP protein undergoes abnormal metabolism and processing, resulting in the production of Aβ. Studies have shown that astrocytes carrying APOE4 can provide excessive cholesterol to neurons, promoting the expansion of lipid rafts, accumulation of APP, and the formation of Aβ [41]. γ-secretase, located on lipid rafts, can cleave APP into Aβ. When corticotropin-releasing factor, a stress response mediator, is elevated, γ-secretase is upregulated, which further increases Aβ secretion [42]. Drugs targeting lipid raft formation, such as platelet-activating factor antagonists, can enhance the intracellular degradation of Aβ [43]. Individuals with AD display higher levels of acid sphingomyelinase and acid ceramidase, resulting in increased concentrations of sphingolipids [44]. A study comparing sphingolipid concentrations in the cerebral cortex of AD patients and individuals without the disease revealed that AD patients have elevated basal levels of long-chain sphingolipids C22:0 and C24:0 [45]. Moreover, alterations in the levels of sphingolipids have been observed in individuals diagnosed with idiopathic PD, indicating a potential association between sphingolipids and the development of PD [46]. In a mouse model of PD with glucocerebrosidase mutation, the inhibition of sphingolipid synthase activity led to a reduction in the buildup of insoluble α-synuclein oligomers and ubiquitinated proteins, providing additional evidence for the connection between PD and sphingolipids [47].

The correlation between the accumulation of Bis(monoacylglycero)phosphate (BMP) in late endosome-lysosome compartments and neurodegenerative diseases has been established through research. BMPs are localized within the inner membranes of late endosomes (multivesicular bodies) and lysosomes, where they contribute to the multivesicular/lamellar morphology of the endolysosomal network. BMPs are elevated in cell types such as macrophages that rely heavily on lysosomal function [48]. In the case of AD, the upregulation of the lipid kinase Vps34, which is responsible for phosphatidylinositol 3-phosphate synthesis, leads to BMP accumulation. Similarly, in individuals carrying the LRRK2 G2019S mutation (the most common genetic determinant of PD identified to date) and experiencing cognitive decline, BMP accumulation is associated with the onset of PD [49].

Individuals diagnosed with HD and ALS display disruptions in the metabolic pathways of sphingolipids and cholesterol within the brain. Specifically, the HD group exhibited elevated levels of sphingosine-1-phosphate lyase 1 and reduced levels of sphingosine kinase 1 in the striatum and cortex compared to the normal group. Individuals with HD display heightened levels of cholesterol ester (CE) in both their tail and shell nuclei. This finding suggests that the impaired synthesis of cholesterol during the development and repair of the myelin sheath in the CNS could contribute to the pathogenesis of ALS. It is noteworthy that the composition of myelin phospholipids in SOD1 G93A rats is altered, indicating a potential compromise in lipid production by oligodendrocytes in ALS patients [50].

The protein ApoE plays a crucial role in facilitating the transportation of lipids in both the brain and periphery. Polymorphism in the APOE gene has been identified as potential risk factors for cellular dysfunction, which can result in abnormalities in calcium signaling, energy metabolism, and lipid metabolism. The relationship between APOE variants and the manifestation of AD and other proteinopathies has garnered significant scholarly attention in recent times. APOE ε2 is considered neuroprotective, whereas APOE ε4 is considered a risk factor for AD, relative to the common ApoE ε3 allele. However, these variants show different behavior, such as their main interactors (LRP1 for ApoE2 and VLDLR for ApoE4), efficiency of intracellular lipid transport (higher for ApoE2, because of the weaker bind low density lipoprotein receptor (LDLR)) or affinity for Aß (reduced for ApoE4, because of VLDLR’s lower rate in internalization of APOE–Aβ complex) [51]. Furthermore, there has been a growing scholarly interest in investigating the impact of aberrant lipid metabolism in astrocytes on the progression of neurological disorders, such as AD [52, 53].

### Abnormal lipid metabolism of astrocytes in AD

The significance of FAs and cholesterol in astrocytes in the context of AD is noteworthy. Neurons prevent lipotoxicity by transferring their excess intracellular FAs to astrocytes via ApoE, which is transported intracellularly by binding to fatty acid-binding proteins (FABPs) and primarily stored in LDs. Subsequently, FA can be transported to mitochondria for β-oxidation. However, the accumulation of LDs caused by dysfunctional mitochondria in astrocytes and excessive FA uptake can lead to lipid metabolism disorders.

Elevated concentrations of free FAs have been recognized as a potential factor in the development of neuroinflammation, consequently heightening susceptibility to AD. Conversely, cholesterol assumes a pivotal function in synaptic physiology, as it is either synthesized by neurons or conveyed from astrocytes to neurons. Specific cholesterol metabolites, including 24-hydroxycholesterol, have demonstrated anti-inflammatory characteristics.

In AD, the presence of intracellular cholesterol buildup in astrocytes has the potential to induce lipotoxicity. Additionally, ApoE plays a crucial role as the principal carrier for lipids, encompassing FAs and cholesterol, and its ApoE4 subtype can contribute to the progression of AD through various lipid metabolic pathways.

#### Fatty acids (FAs)

Astrocytes obtain FAs from both exogenous and endogenous origins. Astrocytes depend on the BBB for the delivery of FAs originating from extracerebral sources. Certain FAs have the ability to permeate the BBB and be absorbed by astrocytes within the brain. Foreign substances have the ability to penetrate the brain and be absorbed by astrocytes in the form of complexes with transport proteins, like FABP, which can traverse the BBB.

Glucose and free FAs have a direct effect on neurons in the hypothalamic periventricular nucleus, which can be modulated by ketones released by astrocytes. This suggests that brain ketone levels play an important role in the uptake of exogenous FAs by astrocytes [54]. Astrocytes also have the ability to detect peripheral FAs. A decrease in the leptin pathway caused by a high-fat diet reduces the coverage of astrocytes on Pro-opiomelanocortin (POMC) neurons and increases the synaptic connections between POMC and Agouti-related peptide (AgRP) neurons, but does not affect the number of astrocytes [55]. In vitro experiments have shown that polyunsaturated fatty acids from outside the brain enter astrocytes after passing through the endothelial cell monolayer and lower layer [54]. Additionally, LDL can be engulfed by endothelial cells and regulated by astrocytes [56]. The engulfed FAs can be released by astrocytes in an HDL-like manner and transported to neurons and other glial cells. LPL, expressed in hypothalamic astrocytes, promotes the uptake of cellular lipids and lipoproteins through hydrolysis and non-hydrolysis pathways. The expression of LPL is reduced after exposure to triglycerides, resulting in a decrease in LD, which may indicate impaired uptake of lipids from peripheral, glial, and neuronal sources, potentially affecting neuronal toxicity [57]. Conversely, the specific knockout of the LPL inhibitor ANGPTL4 inhibits mitochondrial degeneration in astrocytes caused by a high-fat diet, thereby maintaining the efficient processing of FAs by astrocytes [58].

Butyrate plays a crucial role in promoting fatty acid oxidation. In the presence of succinate and ketone bodies, butyrate significantly increases respiratory state 3 [1, 59]. This enhances the efficiency of mitochondria and makes it more challenging to produce reactive oxygen species (ROS). Furthermore, butyrate is converted to butyryl-CoA through acyl-CoA synthetase short-chain family member 2 (ACSS2), which antagonizes the binding of the metabolic intermediate (MCoA) to inhibit CPT1A, upregulates CPT1A activity, and promotes fatty acid oxidation [60]. When the butyrate pathway is impaired, hippocampal mitochondrial function can be compromised through HDAC4, leading to the downregulation of synaptic proteins [61] Butyrate sodium is the sodium salt form of endogenous butyrate, which is formed by the fermentation of dietary fibers by probiotics in the colon. It exerts anti-inflammatory effects in the brain by inhibiting the production of MCP-1, IL-1β, and CXCL10 [62, 63]. Additionally, the inhibitory effect of butyrate on brain inflammation shows gender differences, with butyrate exerting anti-inflammatory effects in female astrocytes but not in males [64].

Astrocytes have the capacity to uptake FAs that originate within the brain not only from the cerebrospinal fluid via the BBB but also from neighboring cells, including neurons and oligodendrocytes. In instances of heightened FAs accumulation within overactive neurons, lipoprotein ApoE-containing particles are conveyed out of the cells to astrocytes to avert lipotoxicity and are subsequently internalized by astrocytes [65]. This phenomenon could be ascribed to the elevated expression of genes in astrocytes in contrast to neurons [66]. Moreover, FAs absorbed by astrocytes undergo degradation to generate ATP and are subsequently excreted into the extracellular environment upon stimulation by glutamate released from neurons. This process triggers the activation of intermediary neurons, ultimately resulting in heightened synaptic inhibition. In the absence of astrocytes, neurons have a tendency to accumulate excessive FAs in LDs, which can result in lipotoxicity and mitochondrial dysfunction [67]. Astrocytes can also obtain FAs through the process of engulfing cellular debris within the brain.

However, when the ApoE4 gene, which is associated with AD, is expressed in the brain, the ability of astrocytes to effectively clear FAs is diminished, resulting in their accumulation. As a consequence, astrocytes become overwhelmed and can only reduce the uptake of FAs from neurons.Moreover, the ApoE genotype exerts an impact on neuronal lipid metabolism, as ApoE4 neurons demonstrate a reduced ability to generate LDs in comparison to ApoE3 neurons. Consequently, this leads to the accumulation of FAs within the cytoplasm, posing a potential threat to mitochondria [67].

Exogenous FAs have been identified as a prospective alternative carbon source for neurons in the management of CNS metabolic disorders [68]. Upon intragastric administration of butyrate, a ketone body, to mice with AD induced by intracerebroventricular injection of Aβ, it was observed that butyrate entered astrocytes through MCT1. Further investigation revealed that butyrate facilitated fatty acid oxidation in astrocytes, thereby serving as an energy source [69]. Medium-chain fatty acids are transported into cells through diffusion and FATP, and subsequently undergo beta-oxidation in the cytoplasm and ketogenesis in mitochondria to provide supplementary carbon sources. In the CNS, astrocytes play a crucial role in the metabolism of long-chain fatty acids (LCFAs). This process involves the internalization of LCFAs through APOE and their binding to acyl-CoA binding protein, resulting in the formation of triglycerides and phospholipids. Recent studies have shown that astrocytes expressing ApoE4 exhibit decreased uptake of palmitate, particularly saturated palmitate, compared to those expressing ApoE3 [67]. Additionally, the intracellular transportation of polyunsaturated fatty acids relies on FABP.

FABP are a group of small intracellular proteins (14–15 kDa) that play a crucial role in the transportation of FAs to the endoplasmic reticulum for signal transduction and membrane synthesis, as well as to the nucleus for lipid-mediated transcriptional regulation [70]. In adult mammals, the FABP family comprises 10 members, including FABP7, FABP5, and FABP3. FABP7, predominantly found in astrocytes, forms LDs to provide protection against reactive oxygen species (ROS) toxicity, and its mRNA expression exhibits synchronized oscillation with the sleep–wake cycle.A proteomic screening study of postmortem AD brains found that FABP7 was upregulated in the brains of patients with symptomatic AD compared to those with asymptomatic AD. Sleep disorders can manifest several years before cognitive impairments in AD patients [71]. This is attributed to the reciprocal relationship between fragmented sleep and Aβ accumulation [71, 72]. These findings suggest that the upregulation of FABP7 in astrocytes may occur early in the progression of AD.

After exogenous lipid molecules enter the cell, FAs are released and combine with glycerol molecules to form triglycerides. This process typically occurs on the smooth andoplasmic reticulum membrane.Triglycerides are transported into the cytoplasm and regulated by lipid droplet-associated proteins, such as perilipin 1, to form small LDs. LDs are composed of an oily core enveloped by a lipid droplet membrane and serve as storage sites for lipids. When the number of LDs increases, fusion between droplets can occur, leading to the formation of larger droplets. The growth and fusion of LDs are regulated by various lipid droplet-associated proteins, such as the lipid droplet fusion protein (e.g., TIP47). When cells require energy, LDs can be degraded, releasing FAs. This process occurs in the mitochondria of the cytoplasm through the β-oxidation pathway, which breaks down FAs into acetyl-CoA and generates energy. During fluctuations in cellular fatty acid levels, the fatty acid sensor protein CD36 and FABPs can regulate the formation and utilization of LDs.

LDs play a crucial role in the storage of FAs within astrocytes. Through genomic analysis, it has been discovered that astrocytes containing LDs exhibit upregulation of various genes, including GPX8 (which prevents the toxicity of free FA), SOD1 and SOD3 (which neutralize superoxide radicals), FABP5 and FABP7 (which participate in FA transport), and ACSBG1 and DBI (which participate in FA metabolism). Furthermore, it has been observed that astrocytes can metabolize LDs through mitochondrial oxidative metabolism and reduce the levels of ROS when stimulated by NMDA from neurons.

Moreover, the release of glutamate by neurons elicits the release of ATP from astrocytes [73]. The subsequent activation of interneurons by the released ATP induces synaptic inhibition [74]. Given that astrocytes possess the ability to convert neuronal FAs into ATP, it can be deduced that active neurons not only evade the risk of lipid toxicity via FA transport, but also stimulate inhibitory interneurons through the astrocytic release of ATP, thereby establishing inhibitory feedback on themselves. In patients with AD, astrocytes experience a reduction in the uptake of FA from neurons as a result of LD lipid toxicity, which arises from the excessive accumulation of FA within astrocytes. Neuronal FAs are unable to be transported out of the cell, and the metabolic capacity of astrocytes to process FAs is inadequate to sustain regular physiological processes. This finding implies that the formation of LDs has a twofold impact, simultaneously providing neuroprotection and exacerbating AD.

Intracellular FAs can be transported to LDs and subsequently undergo beta-oxidation within the mitochondria. Accumulation of a large amount of FAs inside cells can lead to the generation of peroxides under oxidative conditions. When the amount of peroxides generated exceeds the cell’s ability to clear them, the cell becomes damaged, resulting in oxidative stress. This further exacerbates the generation of peroxides and leads to neuroinflammation. This process is closely linked to the reduction ROS and lipid peroxidation levels in astrocytes. Specifically, astrocytes metabolize FAs, particularly medium-chain fatty acids, through beta-oxidation within the mitochondria. This metabolic pathway leads to the partial conversion of these molecules into ketones or shorter chain fatty acyl-CoA, which are subsequently released [75].

The ketones that are released, including beta-hydroxybutyrate and acetoacetate, have the potential to function as alternative energy sources for neurodegeneration and are actively transported into neurons through the GLUT3 transporter. This mechanism effectively alleviates the detrimental effects of FAs on neurons, highlighting the significant involvement of astrocytes in this process. Additionally, monoacylglycerol lipase plays a pivotal role in linking astrocytes and inflammation during the lipolysis process. Monoacylglycerol lipase exhibits the ability to catalyze the hydrolysis of triglycerides into free FAs and glycerol, thereby facilitating energy production. Additionally, monoacylglycerol lipase can hydrolyze 2-arachidonoylglycerol to modulate the signal transduction of the endocannabinoid system. Experimental evidence has demonstrated that the deactivation of monoacylglycerol lipase in astrocytes effectively impedes the inflammatory response of microglia [76].

Glial cells can regulate fatty acid oxidation and mitochondrial integrity through the GLP-1 receptor signaling. In the absence of GLP-1 receptors, mitochondrial dysfunction and cellular oxidative stress can be induced due to increased FGF21 [77]. In the context of AD pathology, GLP-1 can enhance aerobic glycolysis in glial cells through the PI3K/Akt pathway. This promotes fatty acid oxidation, reduces ROS generation, and further enhances support for neurons [77]. Studies have found that Aβ oligomers inhibit the PI3K-Akt pathway, leading to neuronal death. Insulin is also a crucial regulatory factor for glial cell fatty acid metabolism. It can increase the breakdown of FAs by stimulating the generation of long-chain fatty acyl-CoA. Additionally, enzymes related to fatty acid oxidation and breakdown, such as long-chain fatty acyl-CoA synthetase, very long-chain fatty acid elongase, and acetyl-CoA carboxylase 1, can enhance insulin expression. It is worth noting that insulin can also affect cellular energy metabolism efficiency through the PI3K/Akt pathway.

The importance of APOE in the degradation of FAs is notable. In patients with AD, APOE4 has been observed to hinder the degradation of FA in astrocytes [78], possibly due to its negative effect on astrocytic autophagy. Additionally, APOE4 has been shown to reduce the effectiveness of fatty acid oxidation, particularly for exogenous FAs such as oleate and palmitate, which are primarily saturated.

This phenomenon can be attributed to the upregulation of dynamin-related protein 1 (Drp1) by APOE4, which impedes the fusion of mitochondria and the beta-oxidation of FAs. ApoE has been demonstrated to enhance the expression of genes involved in the metabolism of phosphorus-containing compounds, which are associated with lipid metabolism, including CROT, LPGAT1, and PLPP3. Astrocytes containing ApoE4 exhibit a decrease in both ApoE protein and mRNA levels, as well as a reduction in their capacity for glucose metabolism [79].

The aforementioned reduction leads to a decline in the influx of acetyl-CoA into the tricarboxylic acid (TCA) cycle and a concomitant elevation in lactate synthesis [80]. These astrocytes demonstrate an augmented presence of smaller FAs, a more pronounced inhibition of carnitine palmitoyltransferase-1, and a notable reduction in the uptake of the saturated fatty acid palmitate [67].

Astrocytes serve as the predominant cellular class accountable for metabolic processes involving LCFAs, leading to the generation and dissemination of unsaturated LCFAs (particularly oleic acid esters) and polyunsaturated fatty acids, such as arachidonic acid (AA) and docosahexaenoic acid esters [81].

The metabolic byproduct of AA plays a crucial role in establishing a link between abnormal fatty acid synthesis in astrocytes and the development of AD. Epoxyeicosatrienoic acids (EETs) and epoxydocosapentaenoic acids (EDPs) exhibit anti-inflammatory and neuroprotective properties through various mechanisms, such as the suppression of proinflammatory molecules. Epoxide Hydrolase 2 (Ephx2), the gene responsible for encoding soluble epoxide hydrolase (sEH), is primarily localized within the lysosomes of astrocytes [82]. The rapid metabolism of EETs and EDPs into their respective diols by sEH enhances the potential for inflammation in AD. The elevation of 14, 15-EET, the production of neurotrophic factors in astrocytes, and the regulation of microglial activation can be accomplished by inhibiting sEH activity or deleting the Ephx2 gene. The upregulation of sEH expression in AD leads to a reduction in EETs and EDPs in astrocytes, an increase in the secretion of proinflammatory cytokines, and the initiation of neuroinflammation [82].

The decrease in specialized proresolving lipid mediators (SPMs) connected to AA in individuals with AD is associated with the activation of astrocytes and subsequent neuroinflammation. Resolvins and lipoxins, which are SPMs derived from ω-3 and ω-6 FAs such as docosahexaenoic acid, eicosapentaenoic acid, and AA, play a pivotal role in facilitating the resolution of inflammation. Moreover, it has been observed that SPMs can serve as inhibitory signals by selectively binding to receptors on glial cells and neurons, thereby promoting the elimination of proinflammatory signals and mitigating harm in inflammatory disorders [83]. Recently, there has been a hypothesis suggesting that the failure of SPM-mediated resolution of inflammation within the CNS may contribute to prolonged neuroinflammation and exacerbate the pathology of AD [84]. A previous study has provided evidence that the concentration of lipoxin A4 (LXA4), a constituent of SPM, in the cerebrospinal fluid (CSF) of patients with AD is lower than that in individuals with mild cognitive impairment. The decrease in LXA4 levels is correlated with the severity of cognitive impairment and the buildup of tau protein [85].

The astrocytes of individuals with AD demonstrate augmented compensatory expression of the receptors for SPM resolvin E1, specifically the leukotriene B4 receptor (BLT1) and chemoattractant receptor 23 (ChemR23), along with heightened levels of the inflammatory marker YKL-40. This observation suggests that astrocytes play a role in the pathogenesis of AD by facilitating myelin formation and white matter damage through the direct transport of FAs to oligodendrocytes [86].

**Fig. 4:**
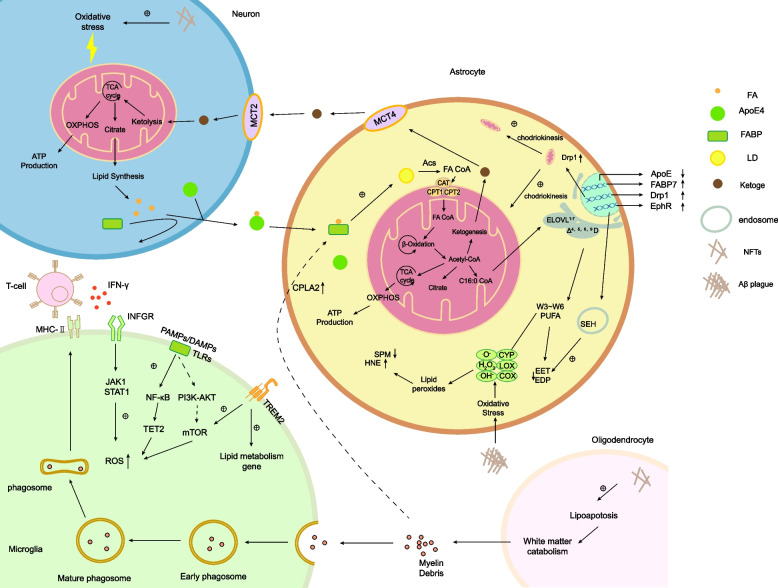
Abnormalities of fatty acid metabolism dominated by astrocytes in Alzheimer’s disease (AD) AD astrocytes are activated by oxidative stress and have abnormal uptake, catabolism and synthesis of fatty acids, resulting in neuronal lipotoxicity. In astrocytes, ApoE4 protein inhibits fatty acid catabolism by inducing the expression of Drp1 (which promotes mitochondrial division) and inhibiting carnitine palmitoyltransferase (CPT), resulting in an increase in intracellular fatty acids and an increase in the formation of fatty acid lipid peroxyl radicals and hydroperoxides. The formation of oxygen radicals and hydroperoxides is increased, including epoxyeicosatrienoic acid (EET), epoxydipentaenoic acid (EDP) and pro-oxidant mediators (SPMs), which inhibit pro-inflammatory signalling, and subsequently increase the inflammatory response of astrocytes. Excess fatty acid excretion from astrocytes induces apoptosis in oligodendrocytes, resulting in myelin debris. Myelin debris activates microglia into a pro-inflammatory phenotype and induces antigen presentation, and also acts as a co-fuel source to increase fatty acid β-oxidation and ketogenesis in astrocytes

The transfer of lipids that are synthesized in astrocytes to oligodendrocytes plays a crucial role in the formation of myelin, as the myelin membrane predominantly consists of glycolipids and phospholipids that heavily rely on FAs as their structural constituents. The synthesis of FAs is imperative for the process of myelin formation in oligodendrocytes, indicating that astrocytes potentially serve as a significant supplier of FAs to oligodendrocytes. The activation of astrocytes and the subsequent release of an excessive amount of saturated FAs by reactive astrocytes are implicated in the toxic effect of oligodendrocyte death [87], which may ultimately result in the demyelination of white matter in AD [88] (Fig. 4).

#### Cholesterol

Recent studies have revealed that the introduction of a high-fat diet to animal models with AD leads to an increased transportation of oxidized sterols to the brain [89]. It observed that specific oxidized sterols possess the capability to initiate inflammation, cellular apoptosis, Aβ production and accumulation, tau hyperphosphorylation, and synaptic impairment, thereby playing a role in the progression of AD [90]. Insufficient research has been conducted on the influence of extracellular oxidized sterols on the functioning of astrocytes. Elevated concentrations of oxidized sterols, induced by Aβ in early or late stages of AD, possess the capability to elicit astrocyte activation and prompt the production of lipocalin-2 (Lcn2) [90]. Lcn2, an acute-phase protein, can bind to the Lcn2 receptors in an autocrine manner and other cells, including neurons, microglia, or oligodendrocytes, in a paracrine manner. Lcn2 exerts an influence on neural morphology and reducing the density of dendritic spines [91]. Lcn2 expression is upregulated through the activation of NF-κB signaling (a pivotal mediator of inflammatory responses) leads to neuronal cell death and additional activation of astrocytes and microglia [92]. In the TGF-α-stimulated cells (include oligodendrocytes), Lcn2 could promote the sustained activation of Epidermal growth factor receptor (EGFR), by inhibiting lysosomal degradation of EGFR and enhancing the localization/recycling of EGFR back to the cell membrane [93]. Targeting PI3K/AKT signaling, which is a key downstream mechanism of EGFR, promotes oligodendrocyte precursor cell (OPC) regeneration and remyelination in an aging context [94].

The oxidized sterols of utmost importance in astrocytes are 24S-OH and 27-OH. Among these, the oxidized sterol 24S-OH is the most prevalent in the brain and is primarily transported from neurons to astrocytes [95]. The brain-specific enzyme cholesterol 24-hydroxylase (CYP46A1) converts excess cholesterol into 24S-OH, which is physiologically expressed in neurons and some astrocytes [91]. Neurons demonstrate an increased ability to express ABCG4, thereby enhancing the efficient export of 24S-OH to astrocytes. Activation of the extracellular liver X receptor (LXR) leads to the upregulation of ATP-dependent ABCA1 expression in neurons and astrocytes. However, insufficient levels of ATP can hinder the efflux of cholesterol from astrocytes to neurons [96]. This mechanism is crucial for maintaining low intracellular cholesterol levels in neurons. Consequently, a decrease in intracellular cholesterol levels in neurons results in a reduction of pThr231Tau/total Tau (tTau) levels in IPSC neurons, thereby alleviating the hyperphosphorylated Tau in AD [97]. The analysis of oxidized sterols in the postmortem brains of individuals diagnosed with AD indicates a significant decrease in 24S-OH levels during the advanced stages of the disease, implying the buildup of cholesterol [89]. This finding suggests that the modulation of astrocytic CYP46A1 expression, which may compensate for the decline in CYP46A1 due to neurodegeneration, could serve as a strategy to mitigate cholesterol accumulation. The release of 24S-OH from neurons induces the activation of ABCA1 and ApoE transcription in astrocytes [95], along with the upregulation of CYP46A1 expression in astrocytes and in close proximity to amyloid plaques [89]. These findings support the notion that lipid metabolism in the brains of AD patients is regulated, aiming to mitigate the buildup of cholesterol. Furthermore, the activation of CYP46A1 appears to have negligible effects on the accumulation of Aβ in the brain. However, the administration of a CYP46A1 activator, specifically EFV, has been observed to improve the behavior of 5xFAD mice. Y-maze test, MWM test and fear conditioning tests were performed, which have shown that EFV treatment improves the spatial long-term memory as well as the short and long-term contextual fear memory but not the spatial short-term working memory or fear cued memory. EFV can increase brain 24S-OH levels, promote cholesterol metabolism in neurons, and enhance specific pre- and postsynaptic proteins [98]. Moreover, recent research has shown that EFV have the potency to activate CYP46A1 to decrease neuronal pTau levels without adversely affecting astrocyte viability [97]. These findings indicate that AD may be caused by insufficient cholesterol conversion, leading to the proliferation of astrocytes.

The cholesterol metabolite 27-OH is found in the highest concentration in plasma and is produced through the catalytic activity of cholesterol 27-hydroxylase (CYP27A1). Due to its lipophilic nature, 27-OH can cross the BBB and enter the brain from the periphery. All tissues have the capability to 27-OH. Under normal physiological circumstances, the concentration of 27-OH in cerebrospinal fluid is directly correlated with its concentration in blood plasma. The brain demonstrates heightened expression of CYP7B1, an enzyme responsible for efficient degradation of 27-OH, in comparison to other organs. Empirical investigations have provided evidence of the brain’s tendency to maintain a diminished level of 27-OH. Astrocytes serve as the primary location for cholesterol synthesis within the brain. 27-OH has the ability to impede cholesterol biosynthesis in the brain through feedback inhibition of the key enzyme 3-hydroxy-3-methylglutaryl-coenzyme A (HMG-CoA) reductase within the cholesterol synthesis pathway [99]. Moreover, 27-OH has the capacity to upregulate ABCA1 and ApoE in the cerebral cortex, thereby facilitating cholesterol transportation. However, in the hippocampus, the upregulation of these proteins is not observed, and the accumulation of Aβ is significantly lower compared to the cerebral cortex. This phenomenon may indicate the presence of a distinct protective mechanism within the brain [100].

Methods aimed at reducing cholesterol biosynthesis, such as the administration of atorvastatin, appear to be beneficial in enhancing lipid metabolism disorders in the brain [101]. Given that cholesterol serves as a precursor for the biosynthesis of various signaling molecules and plays a crucial role in the structure of ion channels and cellular membranes, the inhibition of cholesterol synthesis within the brain could potentially influence signal transduction processes, consequently affecting neuronal excitability. Moreover, cholesterol assumes a crucial function in the glutamatergic signaling pathway, and a reduction in cholesterol levels could potentially lead to the downregulation of glutamate receptors [102, 103]. While cholesterol functions as a negative modulator of presynaptic glutamate release, a decline in cholesterol levels results in a decrease in excitatory postsynaptic currents within glutamatergic synapses. This suggests that the reduction of cholesterol synthesis may mitigate glutamate neurotoxicity [102].

Nevertheless, increased levels of 27-OH have been observed in the brains of individuals affected by early-onset and sporadic AD, and have been associated with mild cognitive impairment in older individuals. According to a study, there is evidence to suggest that CYP27A1 activity is increased in the brain affected by AD, indicating its potential role as a regulator of AD-related neuroinflammation [89]. Notably, 27-OH has the ability to bind and activate LXRα, LXRβ as well as RORγ. 27-OH is also able to bind to additional receptors (e.g., ERα and GPR17). These targets may exert somewhat opposing effects in the same setting.

27-OH, when induced by LPS, can reduce the expression of IL-6 and TNF-α mRNA in LPS-activated microglia and astrocyte co-cultures.However, elevated levels of 27-OH in in vivo models of neuroinflammation can be caused by LPS administration [104]. Human memory is intricately linked to long-term potentiation. Research indicates that 27-OH can increase long-term potentiation by upregulating the level of synaptopodin in the stratum radiatum of the hippocampus, while Oligomeric Aβ has been shown to impede long-term potentiation through CaMKII and lead to dendritic spine loss [105, 106]. Nevertheless, an overabundance of 27-OH inhibits neuronal glucose uptake mediated by the antagonist angiotensin IV by elevating the level and activity of insulin-regulated aminopeptidase [107]. Moreover, 27-OH has the potential to increase the generation of Aβ by upregulating the activity of BACE1 and γ-secretase [108]. Elevated concentrations of 27-OH are toxic to immature oligodendrocytes, while simultaneously facilitating the differentiation of oligodendrocyte progenitor cells [109]. These findings indicate that 27-OH may play a role in regulating glial cell activation and the interaction between neuroinflammation and bioactive lipids.

The process of cholesterol uptake by astrocytes entails the binding and internalization of cholesterol through two principal receptors, specifically the LDLR and the LRP). Research has demonstrated that ApoE, which is secreted by astrocytes, can enhance the degradation of Aβ. Moreover, the interaction between ApoE and LRP4, LDL receptor, and Lrp1 can facilitate the uptake of Aβ by astrocytes, with LRP4 demonstrating superior efficacy at the intracellular level in comparison to the latter two receptors [110]. In individuals with AD, a significant decrease in the level of LRP has been observed in the brain when compared to an age-matched control group. This reduction may be attributed to an increase in Aβ plaques. However, the absence of ApoE does not seem to impact the ability of astrocytes to respond to Aβ [111].

There are two distinct mechanisms by which excess cholesterol is eliminated in the brain. The first mechanism involves the conversion of cholesterol to 24S-OH, which can easily cross the BBB and enter circulation for further metabolism in the liver. The second mechanism involves the esterification of cholesterol through acyl-CoA and cholesterol acyltransferase 1 (including Acetyl-CoA acetyltransferase 1 and Sterol O-Acyltransferase 1) to produce CEs, which are stored in LDs. As previously stated, lipids contained within LDs have the ability to undergo β-oxidation metabolism within mitochondria. The esterified form of total cholesterol in the brain constitutes only a small fraction of 1%, suggesting that the alternative metabolic pathway is not the primary mechanism for cholesterol clearance in the brain [95]. The initial pathway is primarily facilitated by the neuron-specific CYP46A1, while the latter pathway demonstrates higher activity in astrocytes than in neurons, especially in the absence of ApoE or in the presence of excessive exogenous cholesterol [112]. Consequently, it can be inferred that in cases of elevated cholesterol accumulation in the brain, the brain will activate the secondary pathway for cholesterol clearance through astrocytes.

The consumption of cholesterol and disruption of lipid rafts in the brain have been identified as potential mechanisms contributing to aging and AD [113]. In AD, there is a decrease in the efflux of astrocytic cholesterol and an increase in its synthesis [114].

The excessive intake of exogenous cholesterol can activate astrocytes, thereby triggering neuroinflammation mediated by reactive astrocytes in AD. Astrocytes have been observed to potentially augment cholesterol degradation, while simultaneously exhibiting intracellular cholesterol accumulation. The introduction of exogenous cholesterol overload can induce astrocyte activation, increase APP content, and subsequently enhance Aβ synthesis. This phenomenon has been associated with the enrichment of GM1-cholesterol in astrocyte membranes and amplified production of ROS [115].

The involvement of ion channels linked to cholesterol in astrocyte activation in AD may be influenced by the concentration of cholesterol in the cell membrane. P2X7R is a member of the P2X receptor (P2XR) family of ligand-gated cation channels, consisting of three subunits that exhibit a low affinity for extracellular ATP [116]. The functioning of the P2X7R is affected by the levels of cholesterol in the cell membrane, with a decrease in cholesterol content enhancing P2X7R activity and modifying the gating mechanism of ion channels associated with this receptor. The activation of P2X7R has been found to enhance the activity of lipid metabolic enzymes, including phospholipase and sphingomyelinase, thereby influencing the lipid raft composition [117]. Additionally, the persistent activation of P2X7R in astrocytes has been implicated in the potential initiation of Aβ formation [116], which could be linked to inflammatory activation caused by disturbances in cholesterol synthesis. Notably, the upregulation of sterol regulatory element-binding protein cleavage-activating protein (SCAP) can activate the NOD-like receptor thermal protein domain associated protein 3 (NLRP3) inflammasome in mice [118]. Activation of the NLRP3 inflammasome is primarily caused by potassium ion efflux induced by the binding of P2X7R and ATP. In the context of astrocytes in AD, abnormalities in SCAP may result in changes in membrane cholesterol levels, leading to the activation of P2X7R and subsequent inflammation through NLRP3. The distribution of cholesterol within cells is influenced by the APOE genotype, with APOE4 astrocytes exhibiting higher levels of cytoplasmic cholesterol aggregates and lower levels of cholesterol in the plasma membrane than APOE3 astrocytes [119].

The activation of P2X7R and subsequent promotion of Aβ formation can be attributed to the reduction in membrane cholesterol. Furthermore, P2X7R is involved in the facilitation of Ca2 + influx through store-operated calcium entry (SOCE), a process that requires cholesterol in different cell types. Research has demonstrated that an imbalance of calcium in glial cells contributes to the development of AD [120]. Moreover, the clustering of lysosomes near the nucleus is facilitated by a decrease in membrane cholesterol distribution, which is caused by APOE4 [119].

The activation of SOCE in APOE4 star-shaped glial cells is primarily attributed to the mobilization of calcium ions (Ca2 +) from lysosomes, indicating a potential role of reduced membrane cholesterol in this process. The APOE4 apolipoprotein modulates the interplay between calcium signaling, organelle dynamics, and plasma membrane function, thereby influencing the calcium response of astrocytes. The activation of astrocytes has been observed as a consequence of the reduction in membrane cholesterol, which subsequently activates P2X7R. This activation of P2X7R triggers the influx of Ca2 + and efflux of K + , both of which are mediated by P2X7R. Ultimately, this cascade of events leads to the release of glutamate and inflammatory factors by activated astrocytes, culminating in excitotoxicity and neurotoxicity.

The presence of the BBB hinders the absorption of cholesterol from circulation by lipoproteins, resulting in the endogenous synthesis of brain cholesterol. The initial step of cholesterol biosynthesis occurs in the andoplasmic reticulum, where acetyl-CoA is enzymatically converted into mevalonic acid, which undergoes a series of reactions leading to the formation of lanosterol. Lanosterol is then converted to cholesterol in various brain cells through distinct metabolic pathways.

Neurons employ the Kandutsch-Russell pathway to obtain cholesterol from 7-dehydrocholesterol, whereas astrocytes employ the Bloch pathway to obtain cholesterol from desmosterol. Throughout the neonatal and adolescent phases, both astrocytes and neurons possess the ability to synthesize cholesterol from acetyl-CoA in the presence of 3-hydroxy-3-methylglutaryl-coenzyme A reductase (HMGCR), which serves as the enzyme that restricts the rate of the cholesterol synthesis pathway.

In adult mice, astrocytes serve as the primary source of brain cholesterol. Newly synthesized cholesterol undergoes rapid transfer from the andoplasmic reticulum to the plasma membrane. The regulation of cholesterol synthesis is controlled by sterol regulatory element-binding protein 2 (SREBP-2), which remains inactive and bound to SCAP on the andoplasmic reticulum under normal circumstances. However, when low levels of cholesterol are detected in the andoplasmic reticulum, SREBP-2 is activated, leading to the release of the N-terminal domain of SREBP-2 by Scap. The translocation of the domain to the nucleus and its subsequent binding to sterol regulatory elements in gene promoter regions facilitate the process of cholesterol synthesis.

MAMs, specialized regions of the andoplasmic reticulum, are essential for regulating cholesterol homeostasis and display lipid raft-like domains. The activation of MAMs can lead to an increase in cholesterol synthesis.

The components of the γ-secretase complex, namely presenilin-1 and presenilin-2, play a crucial role in the processing of APP. These components are found in abundance in the MAMs. Mutations in presenilin-1 and presenilin-2, which are associated with AD, as well as the presence of ApoE4, can result in an increase in MAMs activity in astrocytes [121].

The enzyme DHCR24 has a significant role in controlling cholesterol biosynthesis and has been observed to be downregulated in both aged astrocytes and astrocytes from AD mice. This downregulation process results in a decline in cholesterol synthesis within astrocytes and a decrease in the protein level of cavin1 in the plasma membrane, which is essential for caveolae formation. Hence, the typical development of membrane caveolae is impacted, as evidenced by recent studies indicating that the reduction in DHCR24 prompts the activation of the astrocyte caveolae-mediated Ras/MEK/ERK signaling pathway, leading to the phosphorylation of tau at Thr181, Ser199, Ser262, and Ser396. This process potentially plays a role in the production of hyperphosphorylated tau in AD [17].

The cholesterol levels found within astrocytes possess the capacity to exert an influence on cholesterol synthesis. Under typical physiological conditions, cholesterol is capable of impeding the activity of HMG-CoA reductase, which is situated within the mitochondria, thereby impeding the accumulation of cholesterol. This inhibition represents a pivotal stage in the rate-limiting progression of cholesterol synthesis. This pathway elucidates the significance of 24S-OH as a physiological suppressor of brain cholesterol synthesis, and the attenuation of its functionality has the potential to induce compensatory cholesterol synthesis in the brain [89]. The development of AD entails the disruption of cholesterol synthesis, which is intricately associated with the stress protein heme oxygenase-1 (HO-1). This protein is predominantly regulated by astrocytes in the brain and plays a dual role in cellular defense and exacerbation of oxidative harm. The upregulation of the Hmox1 gene, which encodes HO-1, has been observed in AD. Its overexpression is significantly linked to intracellular cholesterol dysregulation. In 3xTg-AD mice without the AD phenotype, there is no significant correlation between the levels of cholesterol precursors and oxidized sterols and the level of HO-1. However, with the onset of the AD phenotype, the increase in HO-1 leads to an increase in cholesterol secretion [122].

It has been observed that the accumulation of Aβ in the cerebral cortex of individuals diagnosed with AD leads to an upregulation of HMGCR in astrocytes [96]. This discovery suggests that once Aβ deposition reaches a threshold associated with the AD phenotype, the activation of SREBP by HO-1 surpasses a specific threshold, consequently resulting in an augmented secretion of cholesterol.

Astrocytes require the involvement of apolipoproteins (ApoE, ApoJ/Clusterin, and ApoD) and transport proteins to facilitate cholesterol efflux.Subsequently, these apolipoproteins bind to ABC transporters, specifically ATP-binding cassette transporter A1 (ABCA1), upon secretion, to transport cholesterol to the extracellular space. The control of ABCA1 expression is predominantly regulated by the transcription factors LXR and retinoid X receptor (RXR), which specifically bind to specified promoter regions of target genes following activation by oxysterols and desmosterol. ABCA1 plays a vital role in cholesterol efflux in astrocytes, thereby aiding in the lipidation and maintenance of ApoE at a stable level. ABCA1 facilitates the transport of cholesterol from astrocytes to neurons and eliminates Aβ peptides from the brain [96].

The potential for cholesterol accumulation in astrocytes to induce the upregulation of protein expression associated with cholesterol efflux has been observed. However, this does not necessarily lead to an increase in outflow. Additionally, the endogenous agonist of LXR, 24S-OH, has been found to upregulate the expression of LXR target genes, including ABCA1, ABCG1, and ApoE, in astrocytes [123]. Furthermore, it has been observed that exogenous 27-OH facilitates the removal of cholesterol from astrocytes by stimulating the increased expression of ABCA1 and ApoE [124]. Decreased expression of the ABCA1 transporter protein-encoding-gene is considered a risk factor for AD. Notably, amyloid-like alterations in APP predominantly manifest in membrane regions abundant in cholesterol.

Research has provided evidence indicating that a decrease in the lipidation of apoE results in an elevation of Aβ buildup within the brains of mice afflicted with AD [125]. An in vivo study showed that ApoE lipidation varies between isoforms. APOE2–Aβ and APOE3–Aβ complexes were cleared by both VLDLR and LRP1 at the BBB, whereas APOE4 binding to Aβ switched the clearance pathway of Aβ at the BBB from LRP1 to the VLDLR. As VLDLR mediates the internalization of APOE–Aβ complex at a slower rate than LRP1, ApoE4 is the least efficient ApoE form in Aß clearance [51].

Furthermore, the interaction between Aβ and APP with the alpha subunit of the ATP synthase complex in the extracellular milieu hampers the synthesis of extracellular ATP and influences ATP production.The lipidation activity of ABCA1 is reliant on increased levels of extracellular ATP as a signaling molecule. Inadequate extracellular ATP levels impede the ability of ABCA1 to sustain its functionality, ultimately resulting in a decrease in cholesterol efflux.ABCA1 has the most important roles in cholesterol efflux, apoE-lipidation, and the formation of apoE-HDL in astrocytes [96].

The activation of microglia is influenced by astrocytic APOE, as supported by research demonstrating that the absence of astrocytic APOE leads to a decrease in the microglial response to amyloid plaques [126]. Furthermore, the specific elimination of astrocytic APOE4 has been found to reduce microglial phagocytosis of synapses, indicating a potential protective role against neurodegeneration [127]. In the realm of neurodegenerative diseases, the upregulation of APOE4 in microglia leads to a decline in ApoE production, suppression of the APOE-trem2-plcg2 signaling pathway, augmentation of TNF-α concentrations, and acceleration of inflammatory conditions and lipid metabolism dysfunctions [78, 128]. Nevertheless, APOE4 lacks the capacity to prompt microglia to transition into disease-associated states reactive to plaques or facilitate the genesis of amyloid plaques. The upregulated expression of ApoE by microglia in AD may play a crucial role in the preservation of synapses, given the persistent presence of plaques in AD that require the participation of microglial-expressed ApoE. Additionally, astrocytes possess the ability to transport cholesterol to neurons and oligodendrocytes through the utilization of ApoE [129].

To mitigate the potential neurotoxic effects caused by the buildup of intracellular cholesterol, particularly the excessive phosphorylation of Tau, astrocytes have the capability to transmit miRNAs to neurons through ApoE, thereby suppressing the expression of cholesterol synthesis genes within neurons. Specially, when down-regulating miR-126 in astrocytes, neuronal HMGCR levels were significantly elevated [130]. Notably, a decline in ApoE levels has been observed to lead to a reduction in pTau, while the overall tau content remains unaffected in neurons [127]. This implies that endogenous cholesterol exhibits distinct mechanisms compared to exogenous cholesterol in the formation and accumulation of tau and P-tau.

The transfer of cholesterol from astrocytes to the GM1 domain of neurons, occurring in response to a concentration gradient, induces modifications in the function of the GM1 domain and subsequently alters the threshold for APP processing [131]. The secretion of astrocytic cholesterol mediated by APOE4 can promote the formation of lipid rafts on the neuronal membrane, thereby facilitating excessive production of Aβ and the initiation of neuronal amyloidosis [41]. The synaptic vesicle protein, which is responsible for cholesterol binding, is susceptible to the influence of APOE4 and synaptic events [132]. Moreover, there is a decrease in the transfer of cholesterol from astrocytes to neurons, suggesting the occurrence of early hippocampal synaptic loss and functional impairment in AD, ultimately resulting in cognitive decline [132]. It is worth noting that the loss of synapses is the most dependable indicator of cognitive decline.

In neurons, cholesterol metabolism can be enhanced by the action of BDNF, thereby promoting synapse formation and development [133]. At the same time, it also requires astrocytes to provide sufficient cholesterol to promote synapse formation [114]. When the transport of cholesterol from astrocytes to neurons is reduced, damage to synaptic structures in neurons and a decrease in synaptic vesicle release at presynaptic terminals can be observed [134, 135]. Under AD pathology, cholesterol metabolism in astrocytes is impaired, leading to reduced cholesterol transport to neurons, which limits synapse formation in neurons. Disruption of cholesterol metabolism within neurons can also impair synaptic function and neurotransmitter transmission.

Additionally, elevated levels of intracellular cholesterol are essential for the maturation of oligodendrocytes, as they aid in the synthesis of myelin phospholipids [136]. The impact of cholesterol synthesis in astrocytes on the myelination process of both gray matter and white matter is directly influenced by its extent [137]. As individuals age, the decline in cholesterol synthesis in oligodendrocytes results in a greater reliance on astrocytes, facilitated by ApoE, for the supply of myelin phospholipids. In AD, the effect of reactive astrocytes on myelin generation can range from beneficial to harmful, depending on the degree of proliferation. A moderate level of proliferation has been found to promote the proliferation and viability of OPCs through the action of soluble cytokines such as CNTF and FGF2. However, an excessive level of proliferation can lead to the apoptosis of oligodendrocytes, which is facilitated by the excessive release of saturated FAs by TNF-α [87]. The reduction in phospholipids in white matter myelin begins at the age of 40 and becomes more pronounced in individuals with cognitive impairment associated with AD [136].

The clusters of myelin sheaths that have been formed most recently in white matter are highly susceptible to age-related and pathological damage, making them the initial targets of deterioration [138]. This suggests that the degradation of white matter myelin could serve as an early indicator of AD. The impairment of myelinated sheaths leads to the release of sulfatides, which are the predominant lipid components of myelin phospholipids. These sulfatides can be acquired by lipoprotein particles associated with APOE, which are released by astrocytes through a "kiss and run" mechanism. The lipoprotein particles associated with APOE and containing sulfatide undergo metabolic degradation through the endocytosis pathway, referred to as sulfatide depletion [139]. Individuals with AD exhibit a decrease in white matter cholesterol, FAs, myelin basic protein, and myelin protein phospholipids [140]. This occurrence can be attributed to astrocytes breaking down myelin lipid metabolism to generate ketones in response to the brain’s elevated energy demands [141].

**Fig. 5:**
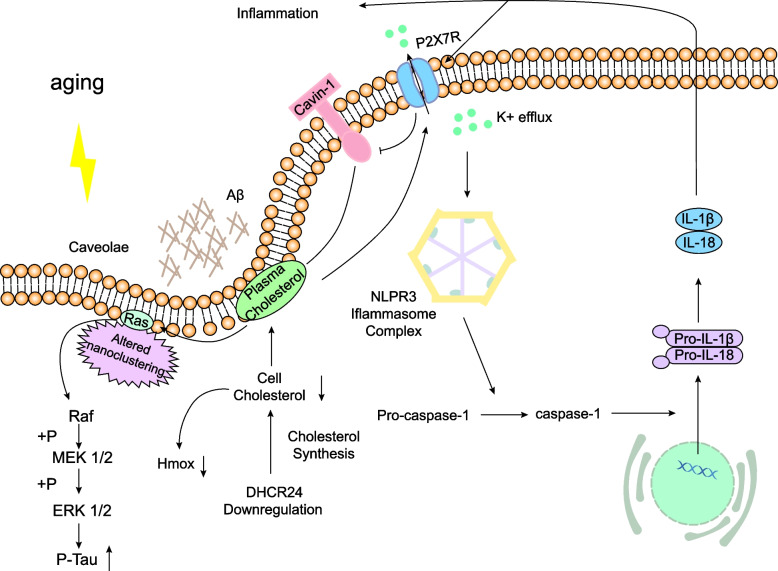
The process of cholesterol biosynthesis is imperative for the effective clearance of Aβ by astrocytes. Nevertheless, the down-regulation of DHCR24, a crucial enzyme in cholesterol synthesis, occurs in astrocytes under aging stimuli. This down-regulation results in a reduction of intracellular and membrane cholesterol content. The downregulation of intracellular cholesterol levels induces the upregulation of the Hmox1 gene and facilitates cholesterol biosynthesis. The decrease in membrane cholesterol content further diminishes the content of membrane Cavin-1, leading to the disruption of caveolae. Consequently, the nanoclustering of Ras is altered, which activates the Raf-MEK-ERK pathway, ultimately leading to an increase in the phosphorylation of Tau protein. Concomitantly, the reduction of membrane cholesterol elicits the activation of P2X7R on the membrane, resulting in the efflux of K + . This decrease in intracellular K + triggers the activation of the NLRP3 inflammasome complex, which catalyzes the cleavage of Pro-Caspase-1 into Caspase-1, thereby facilitating the maturation of Pro-IL-1β into IL-1β and its subsequent release into the extracellular milieu. The extracellular IL-1β can further augment the expression of P2X7R and mediate inflammatory responses

The correlation between the reduction in white matter myelin phospholipids and the genetic susceptibility factor APOE4 [142] for sporadic AD is significant. This particular genetic variant hinders the formation of myelin sheaths by interfering with cholesterol transfer between astrocytes and oligodendrocytes, rather than being a result of amyloid plaque accumulation or neuronal degeneration [136]. Some scholars propose that the activation of astrocytes can lead to increased degradation of myelin, which, in turn, can raise the risk of AD through oxidative stress and neuroinflammation [143] (Fig. 5).

## Conclusions and future directions

The incidence of AD increases with age, making it a common condition among the elderly. AD is characterized by the presence of Aβ and hyperphosphorylated tau proteins in the brains of affected individuals. Astrocytes, which are nonneuronal cells in the brain, play a crucial role in the normal functioning of neurons and the maintenance of neuronal stability, plasticity, and lipid metabolism [18, 36].

The potential influence of astrocyte-related lipid metabolism disorders on the advancement of AD in affected individuals’ brains is noteworthy. These disorders can induce lipotoxicity in neurons, encompassing the toxicity of FAs, thereby hindering the regular physiological functioning of neurons and ultimately culminating in neuronal impairment. According to research, ApoE4 reduces the efficiency of transporting FAs from neurons to astrocytes. It also decreases the ability of astrocytes to break down FAs by affecting the expression and activity of enzymes related to FA metabolism and altering the morphology of mitochondria. This leads to the accumulation of lipids in astrocytes and neurons. The accumulation of lipids can lead to lipotoxicity, oxidative stress, and neuroinflammation. Additionally, the expression of ApoE4 reduces the ability of astrocytes to clear Aβ, which contributes to the pathological development of AD.

This phenomenon is believed to be associated with the development of Aβ and hyperphosphorylated tau, whereby a potential contribution to this process may be attributed to a reduction in astrocyte-derived cholesterol and an elevation in endogenous cholesterol within neurons [17, 116]. The perturbation of lipid metabolism in astrocytes can have a direct impact on neuronal health through disturbances in cholesterol homeostasis, triggering neuroinflammation induced by peroxidation byproducts, and interfering with energy metabolism. Alternatively, it may further contribute to the pathophysiology of AD by impacting astrocytic clearance of Aβ.

The concept of focusing attention on astrocytes [28] and maintaining the balance of two essential lipid components—FAs [68, 69] and cholesterol homeostasis [90, 97], offers new possibilities for therapeutic intervention to hinder or delay the progression of AD. Promising targets for therapy include ApoE4, which has undergone extensive investigation [67, 70], as well as enzymes associated with lipid metabolism, such as monoacylglycerol lipase [68] and CYP46A1 [90]. Notably,the role of butyrate in inhibiting neuroinflammation has received increasing attention in recent years. It is produced by beneficial bacteria in the gut and exerts anti-inflammatory effects when it reaches the brain. Butyrate in the human body can inhibit lipid accumulation, oxidative stress, and neuroinflammation by enhancing the efficiency of mitochondrial fatty acid metabolism, thus suppressing the formation of reactive oxygen species (ROS) and the upregulation of pro-inflammatory factors, thereby inhibiting AD pathology. However, it is worth noting that there are reports suggesting gender heterogeneity in the improvement of mitochondrial metabolism efficiency by butyrate. Therefore, further research is needed to explore the relationship between gender and the impact of butyrate on mitochondrial metabolism efficiency. Dysfunction of CYP46A1 in the primary pathway responsible for cholesterol metabolism can result in increased accumulation of neuronal cholesterol esters, leading to the generation of APP and Aβ. Recent studies have shown that the reduction of cholesterol esters through the action of CYP46A1 can effectively decrease Aβ and tau pathology in isogenic induced pluripotent stem cell (iPSC)-derived neurons, and is found to be better tolerated in comparison to HMGCR inhibitors (statins) [97]. Efavirenz, an activator of CYP46A1, has been shown to enhance cholesterol metabolism in the brains of early-stage AD patients [144]. However, due to substantial gender differences in cholesterol metabolism mediated by CYP46A1 [145], and the safeguarding impact of age-related CYP46A1 upregulation in females rather than males, indicating a pivotal involvement of sex hormones [146], further exploration is warranted to develop therapeutic strategies focusing on CYP46A1.

Additional research is necessary to comprehensively comprehend the interplay between astrocytes and lipid metabolism, specifically concerning the influence of lipid metabolism disorders on astrocyte reactivity, the potential correlation between astrocytes and oligodendrocytes in fatty acid metabolism, and the potential mechanism of signal communication via lipid metabolism between astrocytes and tanycytes in peripheral lipid levels. Although preliminary findings in ApoE drug research have been documented, extensive clinical trials are imperative to substantiate these discoveries [147].

## Data Availability

No datasets were generated or analysed during the current study.

## Abbreviations

AD: Alzheimer’s disease
Aβ: Amyloid-beta
FAs: Fatty acids
CNS: Central nervous system
PTM: Post-translational modifications
MS: Multiple sclerosis
HD: Huntington’s disease
PD: Parkinson’s disease
ALS: Amyotrophic lateral sclerosis
EAAT2: Excitatory amino acid transporter 2
SOD: Superoxide dismutase
BMP: Bis(monoacylglycero)phosphate
CE: Cholesterol ester
FABP: Fatty acid-binding protein
MCT: Monocarboxylate transporter
FATP: Fatty acid transport protein
LCFAs: Long-chain fatty acids
LPL: Lipoprotein lipase
ROS: Reactive oxygen species
TCA: Tricarboxylic acid
Drp1: Dynamin-related protein 1
sEH: Soluble epoxide hydrolase
Ephx2: Epoxide Hydrolase 2
EET: Epoxyeicosatrienoic acid
EDP: Epoxydocosapentaenoic acid
Lcn2: Lipocalin-2
SPMs: Specialized pro-resolving lipid mediators
AA: Arachidonic acid
24S-OH: 24(S)-hydroxycholesterol
27-OH: 27-Hydroxycholesterol
CYP46A1: Cholesterol 24-hydroxylase
CYP27A1: Cholesterol 27-hydroxylase
LXR: Liver X receptor
RXR: Retinoid X receptor
LDLR: Low density lipoprotein receptor
LRP: Low density lipoprotein receptor -related protein
VLDLR: Very low density lipoprotein receptor
APP: Amyloid precursor protein
BBB: Blood–brain barrier
SREBP: Sterol regulatory element-binding protein
SCAP: Sterol regulatory element binding protein cleavage activating protein
NLRP3: NOD-like receptor thermal protein domain associated protein 3
APOE: Apolipoprotein E
SOCE: Store-operated calcium entry
MAMs: Mitochondria-associated endoplasmic reticulum membrane
HMG-CoA: 3-hydroxy-3-methylglutaryl-coenzyme A
HO-1: Heme oxygenase-1
HMGCR: 3-hydroxy-3-methylglutaryl-coenzyme A reductase
ABCA1: ATP-binding cassette transporter A1
GM1: Mnosialotetrahexosylganglioside1
OPC: Ooligodendrocyte precursor cell
